# Effectiveness of School-Based Psychoeducational Program in Reducing Bullying and Improving Self-Esteem: A Systematic Review

**DOI:** 10.3390/healthcare14030330

**Published:** 2026-01-28

**Authors:** Malena Barba Muñoz, José Antonio Zafra-Agea, Eva Martí Marco, Martín Flores-Saldaña, Enrique J. Vera-Remartínez, Aurora Esteve-Clavero, Maria Pilar Molés-Julio

**Affiliations:** 1 Nursing Sciences, University Jaime I of Castellón, 12071 Castelló de la Plana, Spain; 2 Nursing Department, Faculty of Medicine, Universitat Autònoma de Barcelona, 08193 Bellaterra, Spain; 3 Nursing Department, Faculty of Science Health, University Jaime I of Castellón, 12071 Castelló de la Plana, Spain

**Keywords:** bullying, psychoeducational program, self-esteem, social skills, nursing, mental health

## Abstract

**Background/Objectives**: Bullying refers to a specific form of mistreatment that occurs in the school setting and is characterized by intentionality and persistence over time. It should be noted that some elements, such as low self-esteem and lack of social skills, are usually present in both victims and aggressors, so interfering in these aspects can lead to a decrease in the incidence. Thereby, being a victim of bullying is a key factor in the development of multiple mental health issues, such as depression or even suicide. Consequently, mental health nurses play a fundamental role in health education in order to be able to act when necessary and to prevent these types of unfavorable circumstances that can lead to psychiatric disorders. This systematic review aimed primarily to evaluate the effectiveness of psychoeducational programs in reducing school bullying and, secondarily, to analyze their influence on children’s self-esteem. **Methods**: Data were obtained through a comprehensive search of PubMed, Cochrane, and Scielo, following PRISMA guidelines. Studies evaluating evidence-based interventions, including the Olweus Bullying Prevention Program (OBPP), the KiVa Anti-Bullying Program (KiVa), Positive Behavioral Support systems, and standardized social–emotional learning programs, were eligible for inclusion. **Results**: Findings revealed that most interventions showed a positive impact on bullying reduction and self-esteem improvement. However, effectiveness differed depending on contextual factors, such as the educational stage, school climate, cultural setting, and the degree of family involvement, as well as the extent to which each program was adapted to the specific needs of each school environment. **Conclusions**: Psychoeducational programs demonstrate overall effectiveness in reducing bullying behaviors and enhancing self-esteem in children. Nevertheless, outcomes differ depending on school characteristics, cultural context, and the level of family participation, highlighting the need for interventions tailored to each educational setting.

## 1. Introduction

Recent international data confirm that bullying remains a major public health concern worldwide. According to the World Health Organization (WHO), peer violence affects millions of children and adolescents each year, contributing significantly to emotional distress and increased vulnerability to self-harm [1]. Data from the Health Behaviour in School-aged Children (HBSC) study indicate that between 10% and 35% of adolescents in Europe, Central Asia, and Canada have been involved in bullying, with the most recent report showing that approximately one in four adolescents experienced bullying at least once in the past month [2,3]. These figures underscore the persistent prevalence of bullying across different cultural and educational contexts and highlight the urgent need for effective prevention strategies.

Bullying has been classically defined by Olweus as a sustained form of physical or psychological aggression perpetrated intentionally and repeatedly against a person who has difficulty defending themselves [4]. This phenomenon is characterized by an imbalance of power between the aggressor and the victim and involves clearly differentiated roles, including victims, perpetrators, and, in some cases, bystanders [5]. Research indicates that the prevalence of bullying tends to increase from early adolescence, reinforcing the importance of early conceptual clarification and timely intervention [6].

Over the past decades, multiple school-based intervention programs have been developed to address bullying [7,8,9,10,11]. Among the most prominent are the Olweus Bullying Prevention Program [12,13,14] (1992), the KiVa Anti-Bullying Program [15,16] (2006), and whole-school approaches such as Positive Behavioral Interventions and Supports (PBIS) and Social and Emotional Learning (SEL) frameworks. While many of these programs have demonstrated positive outcomes in reducing bullying behaviors [17], evidence remains heterogeneous across educational stages, cultural contexts, and implementation models [18,19,20]. Variability in effectiveness suggests that program outcomes are influenced by contextual and psychosocial factors beyond the intervention itself [7,21].

Self-esteem has been identified as a central psychological factor associated with involvement in bullying, both as a risk factor and as a consequence of victimization or perpetration [22]. Low self-esteem has been linked to emotional distress, social withdrawal, depressive symptoms, and academic difficulties, whereas adequate social skills and assertiveness are associated with greater resilience and healthier peer relationships [23]. Recent studies reinforce this association: Choqueña (2021) found that adolescents with low self-esteem are significantly more likely to experience both victimization and perpetration of bullying, showing a direct and inverse correlation between self-perception and involvement in aggressive school dynamics [24]. Although self-esteem is frequently assessed in studies evaluating anti-bullying interventions, it is often treated as a secondary outcome and has not been consistently synthesized across psychoeducational programs. This lack of focused analysis limits understanding of how interventions influence children’s emotional well-being beyond behavioral outcomes [25]. In addition to these psychological components, bullying dynamics are also influenced by individual, family, cultural, and broader contextual factors, which interact with children’s emotional development and vulnerability. Research indicates that both victims and aggressors frequently exhibit low self-esteem, which can act both as a vulnerability factor and as a consequence of repeated victimization [22,23]. This underscores the need for interventions that address not only aggressive behaviors but also the underlying psychological determinants that contribute to bullying dynamics.

Despite the availability of diverse intervention models, gaps remain regarding their consistent effectiveness and the involvement of health professionals in their implementation. Nurses, particularly in school and community settings, are strategically positioned to design, deliver, and evaluate psychoeducational programs aimed at preventing bullying and promoting children’s emotional well-being.

Therefore, this systematic review was guided by the following research question: Are psychoeducational programs effective in reducing school bullying and improving children’s self-esteem?

In this context, nursing professionals—particularly those working in school and community settings—are strategically positioned to contribute to bullying prevention through health education, early detection, and the promotion of emotional well-being. Despite the growing implementation of psychoeducational programs, there is a need for a systematic synthesis that integrates evidence on both bullying reduction and self-esteem outcomes from a health-oriented perspective. Therefore, this systematic review aimed primarily to evaluate the effectiveness of school-based psychoeducational programs in reducing bullying and, secondarily, to analyze their impact on children’s self-esteem.

## 2. Methods

### 2.1. Study Design

This study is a systematic literature review, conducted in accordance with the PRISMA 2020 guidelines [26]. The systematic review was retrospectively registered in PROSPERO, CRD420251229949.

### 2.2. Eligibility Criteria

Studies published in English or Spanish were eligible if they evaluated psychoeducational interventions targeting bullying reduction as the primary outcome, reported self-esteem as a secondary outcome, and fulfilled the predefined eligibility criteria (Table 1).

Although randomized controlled trials represent the highest level of evidence for evaluating intervention efficacy, observational studies and systematic reviews were also included to capture contextual factors, implementation characteristics, and real-world effectiveness of psychoeducational programs. This approach is particularly relevant in school- and community-based health interventions, where program outcomes are influenced by educational settings, cultural contexts, and levels of family and institutional involvement.

### 2.3. Information Sources

The databases PubMed, Cochrane, and SciELO were consulted due to their complementary coverage of health sciences, education, and psychoeducational research. PubMed and Cochrane provide access to high-quality biomedical and public health literature, including intervention studies and systematic reviews, while SciELO ensures the inclusion of relevant research from Spanish- and Portuguese-speaking countries, where school-based psychoeducational interventions and nursing-led programs are frequently developed and implemented.

Articles published between 1 January 2020 and 1 January 2025 in English or Spanish were included. In addition, a limited number of earlier studies were considered when they met predefined criteria of scientific reliability, including publication in peer-reviewed journals, evaluation of well-established psychoeducational programs, which constitute the foundational evidence base for many contemporary school-based bullying prevention programs and continue to be implemented in current educational settings, use of validated outcome measures related to bullying behaviors, and clear methodological reporting. This exception was applied consistently to ensure an adequate assessment of long-standing intervention models.

### 2.4. Search Strategy and Evidence Selection

The literature search was conducted using keywords and Boolean operators. The operator “AND” was applied to combine relevant terms (e.g., *Bullying AND Self-Esteem*, *Psychoeducational Program AND Bullying*), while “NOT” was used to exclude unrelated topics such as cyberbullying (*Bullying NOT Cyberbullying*). Additionally, to broaden the search, the operator “OR” was employed, as well as combinations of all of them:

(“*school bullying*” *OR* “*school violence*”) AND (“*psychoeducational program*” *OR* “*social and emotional learning*” *OR* “*PBIS*” *OR* “*KiVa*” *OR* “*Olweus*”) AND (*self-esteem*) AND (*preschool OR primary OR secondary*).

Cyberbullying was excluded from this review due to its distinct conceptual framework, contextual characteristics, assessment instruments, and intervention strategies. As the present review focuses on school-based psychoeducational programs primarily implemented in preschool, primary, and early secondary education, the inclusion of cyberbullying would have introduced conceptual and methodological heterogeneity beyond the scope of the study.

### 2.5. Study Selection Process

Two reviewers independently conducted the selection process in three phases:

1. Screening of titles and abstracts.
2. Full-text review.
3. Application of eligibility criteria.

Discrepancies were resolved by consensus or with the participation of a third reviewer. The entire selection process was documented in a PRISMA flow diagram, detailing the records identified, duplicates removed, studies excluded, and the final number of studies included.

### 2.6. Data Extraction

A standardized template was used to collect the following information: author, year, country, study design, educational stage, sample size, intervention characteristics (duration, number of sessions, family and school involvement), instruments used to assess bullying and self-esteem, and the main outcomes. Data extraction was performed independently by two reviewers.

### 2.7. Methodological Quality Assessment

The methodological quality of the included studies was evaluated using design-specific assessment tools:

- Randomized controlled trials: *Cochrane RoB 2* tool [27].
- Non-randomized or quasi-experimental studies: *ROBINS-I* tool [28].
- Observational studies: *JBI* critical appraisal checklists [29].

Furthermore, the CASPe (Critical Appraisal Skills Programme Español) checklist was applied according to the study type (clinical trials, systematic reviews, or observational studies). The evaluated items included internal validity, external validity, and clinical relevance. Studies with eight or more affirmative responses (≥8) were classified as having high methodological quality.

### 2.8. Data Analysis and Synthesis of Results

A narrative synthesis was conducted, focusing primarily on bullying-related outcomes. Self-esteem outcomes were synthesized descriptively when reported.

Due to the heterogeneity of study designs, contexts, and outcome measures, a narrative synthesis was conducted. The results were organized according to the following:

1. Educational stage (preschool, primary, secondary).
2. Type of program (Olweus, KiVa, PBIS, SEL, peer support).
3. Degree of family and school involvement.

### 2.9. Ethical Considerations

As this study is based exclusively on a review of previously published literature, neither informed consent nor approval from an ethical committee was required. The review process adhered to principles of transparency, methodological rigor, and research integrity. Furthermore, the three reviewers who carried out the study selection, assessment, and synthesis of the evidence are also co-authors of this article, ensuring full accountability and compliance with ethical standards throughout the entire process.

## 3. Results

### 3.1. Characteristics of the Included Studies

During the search and selection process, a total of 16,117 records were initially identified across multiple databases: PubMed (*n* = 16,001), SciELO (*n* = 114), and Cochrane (*n* = 2). Additionally, four records were retrieved from other sources, including cross-references and grey literature. After applying manual filters and removing duplicates, 351 records remained for the screening phase, distributed as follows: PubMed (*n* = 284), SciELO (*n* = 65), and Cochrane (*n* = 2).

Subsequently, the full texts of the potentially relevant studies were reviewed. After applying the predefined inclusion and exclusion criteria, 83 records were considered potentially eligible (PubMed, *n* = 58; SciELO, *n* = 22; Cochrane, *n* = 1; other sources, *n* = 2). At this stage, 182 studies were excluded for not meeting the publication date requirement, and 86 were excluded due to language restrictions.

**Figure 1:**
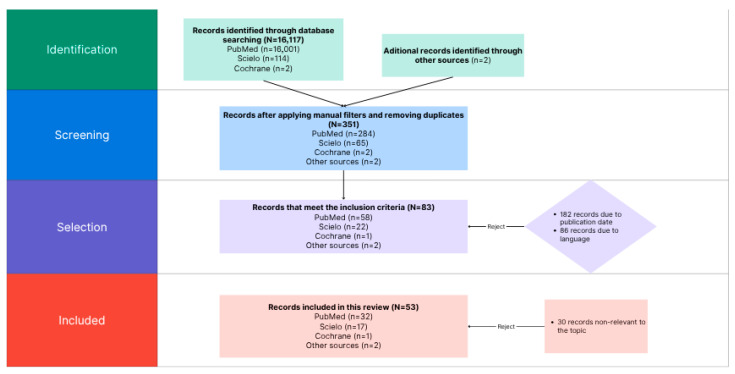
PRISMA 2020 flow diagram for study selection. Source: Authors’ own elaboration based on PRISMA 2020 guidelines.

Finally, 53 studies were included in this systematic review: 32 from PubMed, 17 from SciELO, 1 from Cochrane, and 2 from other sources. In the final selection stage, 30 additional records were excluded for not being directly related to the research topic. This entire selection process is summarized in the PRISMA 2020 [26] flow diagram (Figure 1), which provides a detailed overview of the identification, screening, eligibility, and inclusion phases of the reviewed studies.

### 3.2. Individual Factors

Across the reviewed studies, individual-level factors consistently emerged as key determinants of both bullying involvement and intervention effectiveness, particularly self-esteem, emotional regulation, empathy, and social skills. Several studies agree that low self-esteem, poor emotional intelligence, and deficits in social skills are significant predictors of bullying behavior and its persistence over time [22,23].

Psychoeducational programs integrating Social and Emotional Learning (SEL) [19,20] components demonstrated notable improvements in emotional regulation, empathy, and positive self-perception—key aspects linked to enhanced self-esteem and a reduction in aggressive behaviors. Similarly, interventions emphasizing self-efficacy and conflict resolution, such as the Steps to Respect program [30] and the Shared Concern method [31], have been particularly effective in fostering prosocial beliefs and strengthening self-confidence [32].

Conversely, adolescents with deficits in assertiveness or emotional control tend to be more vulnerable to victimization [33,34,35]. Evidence suggests that both age and educational level influence intervention outcomes, with programs implemented during early stages (preschool and primary education) proving more effective than those applied in secondary education [12,36,37,38].

#### 3.2.1. Assigned Indicators

The most relevant indicators associated with the individual dimension include self-esteem, social self-efficacy, empathy, and emotional regulation. Most of the included studies used validated instruments such as the Self-Esteem Questionnaire for Primary Education (A-EP) [39] and the Peer Victimization Questionnaire (CAI) [40], ensuring consistency in the evaluation of results. Interventions incorporating sessions on self-awareness and emotional skills yielded significant reductions in victimization and improvements in perceived competence, especially among primary school children [41,42].

#### 3.2.2. Factors of Culturation

Several studies emphasized the role of the sociocultural context in shaping the occurrence and persistence of bullying. Programs such as KiVa and Olweus required cultural adaptation for effective implementation across different countries, with notable variations in their outcomes [16,43]. In culturally diverse environments or among ethnic minority groups, limited social and cultural integration may increase vulnerability, highlighting the need for inclusive and context-sensitive approaches. The review by Guevara Benítez et al. (2020) [18] underscores the importance of promoting respect, cooperation, and cultural diversity from early childhood to prevent social exclusion

#### 3.2.3. Psychological Indicators

Psychological indicators are primarily linked to global self-esteem, emotional intelligence, and behavioral regulation. Programs addressing emotional development through structured methodologies reported significant improvements in resilience and subjective well-being. Espinoza-Venegas et al. (2015) [44] demonstrated that enhanced emotional intelligence correlates positively with empathy and peaceful conflict resolution, supporting the hypothesis that reinforcing students’ personal resources contributes to reducing both perpetration and victimization in bullying situations [45].

### 3.3. Family Factors

Family-related variables were identified as significant moderators of intervention outcomes, with parental involvement, emotional support, and home–school consistency emerging as recurrent protective factors [46]. Educational consistency between home and school, open emotional communication, and active parental supervision were associated with lower levels of bullying and improved socio-emotional adjustment [42,47]. Conversely, a lack of emotional support, authoritarian or permissive parenting styles, and exposure to domestic violence are associated with an increased likelihood of aggressive behavior. Studies on PBIS [48,49,50,51] revealed that when families are actively involved in behavioral strategies, the benefits extend to the home environment, reducing conflicts and reinforcing prosocial behaviors.

### 3.4. Community and Social Factors

The community and social environment significantly influence bullying dynamics [47,49,52,53]. Social norms that tolerate violence, low community cohesion, and insufficient supervision in both public and school spaces are well-established risk factors [7,54].

Studies evaluating comprehensive, school-wide, and community-oriented interventions reported positive outcomes in terms of school climate and peer relationships. Programs such as Positive Behavioral Interventions and Supports (PBIS) and KiVa demonstrated consistent reductions in bullying incidents and improvements in perceptions of safety and belonging within the school environment [55].

Similarly, peer-support and peer-mediation programs were associated with strengthened social networks, increased student solidarity, and greater student involvement in prevention activities, contributing to more inclusive and supportive school communities [56,57].

### 3.5. Contextual and Descriptive Evidence on School Bullying

The studies summarized in Table 2 provide contextual and descriptive evidence on the prevalence and impact of school bullying, particularly within the Spanish educational context. Empirical reports and statistical databases consistently document the magnitude of bullying as a persistent phenomenon affecting children’s emotional well-being, social relationships, and academic functioning.

In addition, theoretical and documentary sources included in this subsection emphasize the relevance of integrating bullying prevention into both educational and health-related practice. Collectively, this body of evidence supports the consideration of school bullying as a significant public health concern and highlights the need for coordinated, evidence-based responses across educational, social, and healthcare systems.

Table 3 synthesizes evidence from systematic reviews, meta-analyses, and theoretical works on school bullying prevention and the promotion of self-esteem. Overall, the literature reports positive—though heterogeneous—effects for multi-component, school-wide programs (e.g., KiVa, PBIS, SEL), especially when teacher training and family engagement are integrated. Meta-analyses consistently show small-to-moderate reductions in bullying and victimization, with stronger effects in primary grades and where implementation fidelity is high. Theoretical and conceptual papers converge on ecological, whole-school approaches as the most sustainable strategy.

Table 4 summarizes key methodological guidelines and risk-of-bias tools used to appraise the quality of the included studies. Collectively, PRISMA 2020, RoB 2, and ROBINS-I provide standardized protocols to improve transparency and reduce bias in evidence synthesis and evaluation, while JBI checklists support structured critical appraisal across diverse study designs.

### 3.6. Summary of Empirical and Methodological Findings

A total of 53 empirical and experimental studies were analyzed, encompassing descriptive, quasi-experimental, and randomized controlled designs conducted in Spain, the United States, the United Kingdom, and other countries. These studies examined psychological correlates of bullying and evaluated the effectiveness of school-based psychoeducational interventions across different educational stages.

Overall, interventions incorporating social-emotional learning (SEL) components and Positive Behavioral Interventions and Supports (PBIS) reported improvements in prosocial beliefs, classroom climate, and behavioral outcomes. According to the CASPe checklist, 72% of the included empirical studies were rated as having high or very high methodological quality, whereas descriptive or statistical reports (e.g., AEPAE, 2022; EPData, 2023 [1,6]) were classified as moderate quality due to their limited analytical scope.

Taken together, the evidence synthesized in this review indicates that multi-component psychoeducational interventions addressing emotional education, family engagement, and school-wide participation are consistently associated with reductions in bullying and victimization rates when implemented with adequate institutional support.

## 4. Discussion

The results of this review reveal that school bullying continues to be a highly prevalent and persistent public health issue, exerting a profound impact on the emotional, social, and academic well-being of children and adolescents. The synthesis of evidence demonstrates that interventions focusing on socio-emotional development, family engagement, and community participation are consistently associated with reductions in bullying behaviors and improvements in school climate. These findings are in line with previous systematic reviews and meta-analyses, which report small-to-moderate but consistent effects of multi-component, school-wide programs, particularly when implemented with high fidelity and institutional support. These results are consistent with recent national data from AEPAE (2022) and EPData (2023) [1,6], which confirm the persistence of the phenomenon in Spain and highlight the urgent need for sustained and evidence-based preventive strategies.

At the individual level, psychological and behavioral factors such as self-esteem, emotional regulation, empathy, and social competence play a decisive role in both the onset and persistence of bullying. Studies by León Gualda and Lacunza (2020) and Fernández et al. (2021) [22,23] indicate that higher self-esteem is associated with lower levels of victimization and better social adjustment, while deficits in assertiveness or emotional control increase vulnerability. Likewise, emotional intelligence—evaluated across different educational stages—has emerged as a protective factor against peer violence and a key component of effective interventions. This pattern is consistent with ecological and developmental models, which emphasize that early reinforcement of socio-emotional competencies enhances resilience and reduces susceptibility to peer aggression. Programs integrating socio-emotional learning (SEL) elements have shown significant improvements in empathy, self-awareness, and prosocial beliefs, thereby reducing aggressive and victimizing behaviors.

From a family perspective, the emotional climate of the home, parental involvement, and affective communication have a direct influence on bullying dynamics. Families that establish supportive relationships and reinforce positive behavior tend to promote peaceful conflict resolution and empathy. In contrast, authoritarian, permissive, or neglectful parenting styles, as well as exposure to domestic violence, are linked to higher risks of aggression and victimization. Evidence from PBIS-based interventions further indicates that when families participate actively in behavioral and educational strategies, benefits extend beyond the school context, enhancing emotional well-being and strengthening prosocial conduct at home [48,50]. These findings reinforce evidence from previous reviews indicating that family involvement functions as a key moderator of intervention effectiveness rather than as a mere contextual variable.

At the community and school level, comprehensive and evidence-based programs such as KiVa, PBIS, and SEL have demonstrated consistent effectiveness in reducing bullying prevalence and improving school climate, social cohesion, and collective well-being [11,20]. These initiatives adopt a whole-school approach that fosters cooperation among teachers, students, and families, thereby promoting a culture of respect and inclusion. However, the variability in outcomes reported across studies suggests that effectiveness is strongly influenced by contextual factors such as educational stage, cultural setting, and fidelity of implementation, as highlighted in prior meta-analytic research. Furthermore, interventions based on peer support and mediation have proven effective in empowering students as active agents of change and enhancing their sense of belonging to the school community.

Recent research reinforces the need for an ecological and systemic approach to bullying prevention, recognizing the interdependence between individual, family, school, and societal factors [3,7]. The present findings support this perspective, indicating that interventions addressing a single level in isolation are less effective than those integrating multiple domains simultaneously. The most successful interventions are those that integrate teacher training, parental involvement, peer mediation, and sustained institutional commitment, ensuring that prevention becomes embedded within the school culture rather than confined to short-term initiatives.

Within this framework, the role of school nursing is particularly significant. As frontline health professionals within educational environments, school nurses are well-positioned to identify early signs of victimization or aggression, provide biopsychosocial support, and coordinate interdisciplinary interventions involving educators, families, and mental health services. Beyond their clinical function, nurses can lead emotional education programs, promote self-care, and foster resilience, empathy, and social cohesion among students. This integrative role represents a distinctive contribution of the present review, highlighting nursing not only as a support profession but as an active agent in the design, implementation, and evaluation of school-based psychoeducational interventions.

From a practical and policy perspective, these findings underscore the importance of investing in early, comprehensive, and school-wide prevention strategies supported by stable institutional frameworks. Educational and health policies should prioritize the incorporation of evidence-based psychoeducational programs, interdisciplinary collaboration, and the inclusion of nursing professionals within school health teams. Future research should focus on longitudinal evaluations, standardized outcome measures, and the assessment of implementation fidelity to better understand the mechanisms through which interventions achieve sustainable effects.

Despite the robust evidence supporting these interventions, certain methodological limitations should be acknowledged. The studies reviewed exhibit variability in design, sample size, and measurement tools, which complicates direct comparison of outcomes. Nevertheless, the overall consistency of findings underscores the importance of implementing comprehensive, context-adapted, and periodically evaluated interventions grounded in empirical evidence.

## 5. Conclusions

In conclusion, this review reaffirms the necessity of addressing school bullying through a multidimensional, interdisciplinary, and community-based perspective, in which nursing professionals play a pivotal role in promoting emotional well-being, preventing bullying, and strengthening collaboration between schools, families, and community health networks.

Additionally, it is important to acknowledge that, beyond psychoeducational programs, other intervention models—such as whole-school policies, punitive or disciplinary approaches, peer-support systems, and technology-based tools—have also been implemented to reduce bullying. However, these strategies are associated with relevant challenges.

Limitations: Whole-school interventions require long-term institutional commitment and substantial resources; punitive measures often fail to address the psychological roots of aggressive behavior; peer-support programs depend heavily on adequate student selection and supervision; and digital reporting systems, while useful for detection, lack robust evidence regarding their long-term effectiveness. These limitations highlight the value of psychoeducational interventions, which directly target emotional regulation, social skills, and self-esteem, yet also underscore the need for comprehensive, integrated approaches that combine educational, social, and health-based strategies.

Together, these findings support the implementation of evidence-based, nurse-informed psychoeducational interventions as a cornerstone of sustainable school bullying prevention policies.

## Figures and Tables

**Table 1:** Inclusion and exclusion criteria of the selected studies.

Criteria	Description
Population	Students in Preschool, Primary, and Secondary Education.
Intervention	School-based psychoeducational programs aimed at the prevention and/or reduction of bullying and the enhancement of self-esteem (e.g., *Olweus*, *KiVa*, *PBIS*, *Social and Emotional Learning*, *peer support*).
Comparator	Usual practice, no intervention, or other school-based interventions.
Outcome	Primary: Reduction of bullying (victimization or perpetration) assessed through validated instruments. Secondary: Changes in self-esteem levels, measured using validated scales.
Included designs	Randomized controlled trials, quasi-experimental studies, pre-post studies with or without a control group, and observational studies evaluating interventions, as well as reviews.
Exclusion criteria	Editorials, protocols without results, studies focused exclusively on cyberbullying, research involving university populations, or those not evaluating bullying or self-esteem.

Source: Authors’ own elaboration.

**Table 2:** Summary of empirical, descriptive, and theoretical studies on school bullying.

Author(s) Year	Country	Methodological Design	Type of Source	Study Objective	Main Conclusion	Methodological Quality
AEPAE 2022 [1]	Spain	Descriptive report/statistical data	Empirical	To present the number of severe bullying cases in Spain reported by the SAE.	A total of 11,229 severe bullying cases were documented in Spain, highlighting the magnitude of the problem.	Medium
Cerezo, F. 2009 [4]	Spain	Descriptive study	Empirical	To analyze the situation of bullying in Spanish classrooms.	Bullying represents a significant problem in Spanish schools, with implications for emotional health.	High
Rodríguez López, M.A.; González Fernández, C.T.; Megías Plata, D. 2021 [5]	Spain	Academic handbook	Manual	To address topics in child and adolescent nursing, including school bullying.	Bullying management should be integrated into pediatric nursing practice.	High
EPData 2023 [6]	Spain	Statistical database	Database	To collect updated figures on school bullying.	Data reveal a concerning trend in the prevalence of bullying cases in Spain.	Moderate
Nickerson, A. 2019 [7]	United States	Review/conceptual framework	Theoretical	To propose an evidence-based prevention and intervention framework.	Programs should be evidence-based and implemented comprehensively within the school environment.	High

Source: Authors’ own elaboration.

**Table 3:** Reviews, meta-analyses, and theoretical studies—summary of results.

Author(s) Year	Country	Type	Objective	Main Findings	Methodological Quality
Smith, J.D.; Schneider, B.H.; Smith, P.K.; Ananiadou, K. 2004 [8]	United States	Systematic review	To synthesize evidence on anti-bullying program effectiveness.	Programs show overall positive outcomes with variability across contexts and designs.	High
Miguel, C.; Kilburn, J.; Sanchez, P. 2007 [9]	United States	Meta-analysis	To evaluate the effectiveness of school anti-bullying programs.	Moderate efficacy in reducing bullying; effects depend on program components.	High
Baldry, A.; Farrington, D. 2007 [10]	United States	Systematic review	To analyze the effectiveness of prevention programs.	Several programs are promising, but implementation consistency is lacking.	High
Farrington, D.P.; Ttofi, M.M. 2009 [11]	United Kingdom	Systematic review	To examine school-based programs to reduce bullying and victimization.	Significant reductions in victimization across many programs.	High
Yeager, D.S.; Fong, C.J.; Lee, H.Y.; Espelage, D.L. 2015 [12]	United Kingdom	Multilevel meta-analysis	To assess program efficacy among older adolescents.	Efficacy decreases in older adolescents; age-tailored strategies are required.	High
Nickerson, A. 2019 [7]	United States	Review/conceptual framework	To propose an evidence-based prevention and intervention framework.	Recommends comprehensive, evidence-based, whole-school implementation.	High
Limber, S.P. 2011 [14]	United States	Program review	To analyze the development and evaluation of the Olweus Bullying Prevention Program.	Demonstrated efficacy, with need for updates and contextual adaptations.	Moderate
Uslu, R.; Bağlama, B. 2020 [17]	Turkey	Review of interventions	To evaluate positive behavioral support interventions.	Effective in improving behavior and reducing conflicts.	High
Ttofi, M.M.; Farrington, D.P. 2011 [42]	United Kingdom	Systematic review and meta-analysis	To evaluate school programs to reduce bullying.	Significant reductions, especially with teacher training and family involvement.	Very High
Guevara Benítez, C.Y.; Rugerio Tapia, J.P.; Hermosillo García, Á.M.; Corona Guevara, L.A. 2020 [18]	Mexico	Review	To review social-emotional learning in preschool.	Fosters prevention of behavioral problems and holistic development.	Moderate
Weissberg, R.; Durlak, J.; Domitrovich, C.; Gullotta, T.P. 2015 [19]	United States	Book chapter (review)	To analyze the evolution of social and emotional learning (SEL).	SEL is essential in education with strong empirical support.	High
Durlak, J.A.; Weissberg, R.P.; Dymnicki, A.B.; Taylor, R.D.; Schellinger, K.B. 2011 [20]	United States	Meta-analysis	To evaluate the impact of universal SEL interventions.	Significant improvements in socio-emotional competencies and academic performance.	Very High
Hamodi Galán, C.; Jiménez Robles, L. 2018 [15]	Spain	Review of models	To explore bullying prevention in early childhood education.	Early education is key to preventive measures.	Moderate
Saracho, O.N. 2017 [46]	Germany	Review of strategies	To identify prevention strategies in early childhood education.	Early prevention is fundamental to mitigating later bullying.	Moderate
Rigby, K. 2005 [31]	Australia	Methodological review	To describe and assess the Method of Shared Concern.	Effective in complex conflicts involving aggressors and victims.	Moderate–High
Cowie, H.; Fernández, F.J. 2006 [56]	Spain	Systematic review	To analyze peer-support approaches in schools.	Strengthens cohesion and contributes to reducing bullying.	High
Avilés Martínez, J.M.; Irurtia Muñiz, M.J.; Alonso Elvira, N. 2008 [41]	Spain	Theoretical review	To present structured steps for victim-focused interventions.	Recommends structured strategies to support victims.	Moderate
Robinson, G.; Maines, B. 2012 [53]	United Kingdom	Practical manual/guide	To present a group-support methodology against bullying.	Useful for reducing school conflicts when consistently implemented.	Moderate

Source: Authors’ own elaboration.

**Table 4:** Methodological guidelines and tools—summary of results.

Author(s) Year	Country	Guideline/Tool	Objective	Main Contribution/Application	Methodological Quality
Page, M.J.; et al. 2021 [26]	Spain	PRISMA 2020 (reporting guideline)	To update the Preferred Reporting Items for Systematic Reviews and Meta-Analyses.	Enhances transparency, completeness, and standardization of reporting in systematic reviews.	Very High
Sterne, J.A.C.; et al. 2019 [27]	United Kingdom	RoB 2 (risk-of-bias tool for randomized trials)	To provide an updated framework to assess risk of bias in randomized controlled trials.	Standardizes bias assessment across domains (e.g., randomization, deviations, missing data, measurement, selection of results).	Very High
Sterne, J.A.C.; et al. 2016 [28]	United Kingdom	ROBINS-I (risk-of-bias tool for non-randomized studies)	To evaluate risk of bias in non-randomized studies of interventions.	Offers a rigorous domain-based assessment aligned with causal inference principles.	Very High
Hilton, M. 2021 [29]	Australia	JBI Critical Appraisal Checklists	To provide study-design–specific checklists for critical appraisal.	Facilitates structured quality appraisal tailored to RCTs, quasi-experiments, observational, and qualitative studies.	Very High

Source: Authors’ own elaboration.

## Data Availability

No new data were created or analyzed in this study. Data sharing is not applicable to this article.
